# Taxonomy and phylogeny reveal new species of *Volvariella* (Volvariellaceae, Agaricales) and *Volvopluteus* (Pluteaceae, Agaricales) from eastern India

**DOI:** 10.3897/mycokeys.130.183859

**Published:** 2026-03-24

**Authors:** Entaj Tarafder, Enjamul Hoque, Ang Rinzing Sherpa, Samantha C. Karunarathna, Meimei Wang, Nalin N. Wijayawardene, Qiang Li, Fuqiang Yu, Dong-Qin Dai, Wannapawn Watsuntorn

**Affiliations:** 1 Center for Yunnan Plateau Biological Resources Protection and Utilization & Yunnan International Joint Laboratory of Fungal Sustainable Utilization in South and Southeast Asia, College of Biology and Food Engineering, Qujing Normal University, Qujing 655099, China The Institute of Biotechnology and Genetic Engineering, Chulalongkorn University Bagnkok Thailand; 2 Department of Botany, West Bengal State University, N.24 Parganas, Barasat 700126, West Bengal, India College of Biology and Food Engineering, Qujing Normal University Qujing China; 3 Germplasm Bank of Wild Species & Yunnan International Joint Laboratory of Fungal Sustainable Utilization in South and Southeast Asia, Kunming Institute of Botany, Chinese Academy of Sciences, Kunming 650201, China Kunming Institute of Botany, Chinese Academy of Sciences Kunming China; 4 High-Value Food from Mushrooms and Bioactive Plants in the Green Economy Value Chain Research Group, The Institute of Biotechnology and Genetic Engineering, Chulalongkorn University, 254 Phayathai Road, Pathumwan, Bangkok, 10330, Thailand Department of Botany, West Bengal State University Barasat India

**Keywords:** Basidiomycota, morphological characteristics, phylogenetic analysis, two new species

## Abstract

*Volvariella areolavolvata***sp. nov**. and *Volvopluteus fibrillobrunneus***sp. nov**. are introduced and illustrated from West Bengal, eastern India, based on detailed morphological observations and molecular phylogenetic analyses. Morphologically, *V. areolavolvata* is distinguished by its large-sized basidiomata with a pileus covered by conspicuous silky fibrils, becoming slightly yellowish at the center with age; the presence of a volva with a distinctive, externally areolate cracked surface; oval to ellipsoid basidiospores with a mean value of 7.75 × 4.91 μm; mostly lageniform cheilocystidia, 63–131 × 21–37 μm; and broadly clavate pleurocystidia with a rounded apex, 44–56 × 13–17 µm. *Volvopluteus fibrillobrunneus* is characterized by a pileus with light brown to greyish-brown fibrils; ellipsoid to elongate basidiospores (mean 9.85 × 6.30 μm); mostly clavate cheilocystidia measuring 34–72 × 15–29 μm; and rostrate pleurocystidia with prominent apical appendages, 53–75 × 14–21 µm. Phylogenetic analyses based on combined nrITS and nrLSU sequence data support the recognition of both taxa as distinct species within *Volvariella* and *Volvopluteus*, respectively, forming well-supported clades clearly separated from their closest species. Detailed morphological descriptions, field photographs of the collected basidiomata, comparisons with morphologically similar taxa, and phylogenetic analyses based on the combined nrITS and nrLSU sequence datasets are provided.

## Introduction

The genus *Volvariella* Speg. was established by Spegazzini (1898) with *Volvariella argentina* Speg. as the type species. Members of the genus are widely distributed across temperate, subtropical, and tropical regions (Mehrotra and Aneja 1990). They are mostly found as saprobes on soil, in grasslands, on forest litter, and occasionally on decaying wood. Species of *Volvariella* are characterized by their pluteoid basidiomata, free lamellae, the presence of a membranous volva at the stipe base, a pinkish spore print, smooth and inamyloid basidiospores, and an inverse hymenophoral trama (Singer 1986; Orton 1986; Boekhout 1990; Kaygusuz et al. 2020; Kalichman et al. 2020; Kumla et al. 2022). However, in some studies, *Volvariella* was placed within the family Pluteaceae based on morphological similarities with the genus *Pluteus* (Justo et al. 2011a). Later, molecular phylogenetic studies showed that *Volvariella* s.l. is polyphyletic (Matheny et al. 2006).

Subsequent multi-gene phylogenetic analyses demonstrated that the *Volvariella*-like species with basidiospore lengths shorter than 11 μm and possessing a gelatinous ixocutis-type pileipellis form a distinct clade from *Volvariella* s.str. and are more closely related to the genus *Pluteus* (Justo et al. 2011a). This led to the segregation of the genus *Volvopluteus* Vizzini, Contu & Justo to accommodate these taxa, which are distinct from *Volvariella* s.str. and *Pluteus* s.str. (Justo et al. 2011a, 2011b). More recently, based on six-loci phylogenetic analyses, Volvariellaceae Vizzini, Consiglio & P. Alvarado was introduced to accommodate *Volvariella* s.str. within the order Agaricales (Vizzini et al. 2024). More than 147 species of *Volvariella* have been described around the world (Chattopadhyay et al. 2022; Vizzini et al. 2024; Hussain et al. 2025). To date, 22 species of *Volvariella* have been reported in India (Table 1), of which only eight have been reported from West Bengal, eastern India (Dutta et al. 2011, 2013; Chattopadhyay et al. 2022).

**Table 1.** List of *Volvariella* and *Volvopluteus* species reported from India, showing their state-wise distribution and relevant references.

Species	State	Reference
*Volvariella apalotricha*	Kerala	Pradeep et al. (1998)
*V. bombycina*	Kerala, Rajasthan	Pradeep et al. (1998); Chouhan and Panwar (2021)
*V. bilobata*	West Bengal	Chattopadhyay et al. (2022)
*V. castanea*	West Bengal, Uttar Pradesh,	Massee (1912); Pathak et al. (1978)
*V. cubensis*	Kerala	Mohanan (2011)
*V. delicatula*	West Bengal, Uttar Pradesh	Massee (1912); Pathak et al. (1978)
*V. diplasia*	West Bengal, Uttar Pradesh	Bose (1920a); Rath (1962); Pathak et al. (1978)
*V. earlei*	Rajasthan	Chouhan and Panwar (2021)
*V. glandiformis*	Kerala	Pradeep et al. (1998); Mohanan (2011)
*V. hypopithys*	Kerala, Rajasthan	Pradeep et al. (1998); Chouhan and Panwar (2021)
*V. indica*	Punjab	Kaur et al. (2013)
*V. media*	Tripura	Roy Das et al. (2017)
*V. murinella*	Assam	Gogoi and Prakash (2015)
*V. nigrodisca*	Kerala	Pradeep et al. (1998); Mohanan (2011)
*V. pseudovolvacea*	Kerala	Pradeep et al. (1998); Mohanan (2011)
*V. pusilla*	Kerala, West Bengal, Rajasthan	Pradeep et al. (1998); Dutta et al. (2011); Chouhan and Panwar (2021)
*V. sathei*	Maharashtra	Senthilarasu et al. (2012)
*V. taylorii*	Kerala, Mizoram	Pradeep et al. (1998); Mohanan (2011); Lalrinawmi (2019)
*V. terastia*	Kerala, West Bengal, Uttar Pradesh	Bose (1920b); (1921); Rath (1962); Pathak et al. (1978); Manimohan et al. (1988)
*V. thwaitesii*	West Bengal, Uttar Pradesh	Berkeley (1850); Pathak et al. (1978)
*V. volvacea*	Kerala, West Bengal, Assam, Tripura	Lakshmanan et al. (1979); Pradeep et al. (1998); Florence (2004); Manimohan et al. (2007); Pradeep and Vrinda (2007); Dutta et al. (2011); Mohanan (2011); Gogoi and Prakash (2015); Swargiari and Buragohain (2018); Basumatary and Gogoi (2016); Debnath et al. (2020)
*V. woodrowiana*	Maharashtra, Uttar Pradesh	Massee (1899); Pathak et al. (1978)
*Volvopluteus diversisporus* (Nom. inval., Art. F.5.1)	Punjab	Kaur and Singh (2014)
*Vp. shafferii* (Nom. inval., Art. F.5.1)	Punjab	Kaur and Singh (2014)

The genus *Volvopluteus* is typified by *Volvopluteus gloiocephalus* (DC.) Vizzini, Contu & Justo as the type species. Species of *Volvopluteus* are characterized by medium- to large-sized basidiomata, free lamellae, a pinkish spore print, the presence of a volva at the stipe base, inamyloid, subglobose to broadly ellipsoid basidiospores, and a gelatinous ixocutis-type pileipellis (Justo et al. 2011a; Kaygusuz et al. 2021). To date, only 12 species have been listed in Species Fungorum (www.speciesfungorum.org; accessed on 12 December 2025), viz. *Vp. asiaticus* Justo & Minnis, 2011, *Vp. canalipes* (Murrill) Montoya, Bandala & Esqueda, 2021, *Vp. delicisus* Chuan H. Li & T.H. Li, 2025, *Vp. diversisporus* M. Kaur & Yadw. Singh, 2014, *Vp. earlei* (Murrill) Vizzini, Contu & Justo, 2011, *Vp. gloiocephalus* (DC.) Vizzini, Contu & Justo, 2011, *Vp. longipes* Xue T. Zhu & L. J. Liu, 2025, *Vp. michiganensis* (A.H. Sm.) Justo & Minnis, 2011, *Vp. platensis* Xue T. Zhu & L. J. Liu, 2025, *Vp. shafferii* M. Kaur & Yadw. Singh, 2014, and *Vp. yunnanensis* D.G. Zheng & Karun., 2025. Among these, only two species have been reported from India, viz., *Vp. diversisporus* and *Vp. shafferii* (Table 1). However, both records are currently considered invalid in MycoBank (https://www.mycobank.org; Zheng et al. 2025). Importantly, no species of *Volvopluteus* has been reported from West Bengal in eastern India.

During repeated field surveys conducted from 2024 to 2025 in Barasat, West Bengal, India, several specimens belonging to the families Volvariellaceae and Pluteaceae were collected. Among the new collections, two new species belonging to *Volvariella* and *Volvopluteus* are introduced based on morphological characteristics and combined (nrITS and nrLSU) phylogenetic analyses. Detailed descriptions, illustrations, and phylogenetic analysis results of the two new species are presented.

## Materials and methods

### Sample collection and morphological observation

Fresh basidiomata of *Volvariella* and *Volvopluteus* were photographed in situ at Talikhola and Kazibari, Barasat, West Bengal, India. Collection details were recorded (Shaffer 1957; Boekhout 1990; Rathnayaka et al. 2024), and the specimens were taken to the laboratory at West Bengal State University in plastic collection boxes. Morphological descriptions of the specimens were made following Largent et al. (1977). For color terminology, Kornerup and Wanscher’s (1978) color code was strictly followed. Collected specimens were dried at 45–50 °C with an electric food dryer (Hu et al. 2022).

For microscopic characterization, thin sections from multiple basidiomata were revived in 5% KOH and stained with a 1% Congo red solution (Tarafder et al. 2023; Lu et al. 2024). The notation [30, 2, 1] indicates that measurements were conducted on a total of 30 basidiospores obtained from a multiple-sample collection at a specific time point. Additionally, the abbreviations X_m_, Q, and Q_m_ represent the following: X_mr_, the range of basidiospore arithmetic means of the spore length by spore width (± standard deviation), and X_mm_, the mean of basidiospore means (± SD); Q, the quotient of basidiospore length/width, indicated as a range of variation in n spores measured; Q_mr_, the range of Q_m_ values, and Q_mm_, the mean of Q_m_ values; n, the number of basidiospores measured; and s, the number of specimens involved, with the standard deviation specified. Dried voucher specimens were deposited in the Central National Herbarium (CAL).

### DNA extraction, PCR amplification, and sequencing

Genomic DNA was extracted from the herbarium specimens using the E.Z.N.A.® Fungal DNA Mini Kit (Omega Bio-Tek, Inc., Norcross, USA), with slight modifications to the manufacturer’s protocol and to the protocol described in earlier studies (Tarafder et al. 2022, 2023). After DNA extraction, the primer pairs ITS1 and ITS4 (White et al. 1990; Gardes and Bruns 1993) were used to amplify the internal transcribed spacer (nrITS) gene regions of ribosomal DNA, and LR0R and LR3 (Vilgalys and Hester 1990) were used to amplify nrLSU. The DNA fragments were amplified on an Applied Biosystems 2720 automated thermal cycler, following the methods described by Dutta et al. (2017) and Tarafder et al. (2022). PCR amplification was carried out in a 25 µL reaction volume, which consisted of 12.5 µL of 2× Bench Top™ Taq Master Mix, 9.5 µL of ddH2O, 1 µL of each primer (10 µM), and 1 µL of genomic DNA. The PCR thermal cycling programs for nrITS and nrLSU amplification were as follows: an initial denaturation at 94 °C for 4 minutes, followed by 35 cycles of denaturation at 94 °C for 30 seconds, annealing at 56 °C for 45 seconds, elongation at 72 °C for 1 minute, then a final extension at 72 °C for 7 minutes, and storage at 4 °C. Following amplification, the PCR products were purified with the QIAquick® Gel Extraction Kit (QIAGEN, Germany) and sequenced with the primers listed in Table 2 using the commercial sequencing provider GeneSpec Pvt. Ltd. (Karnataka, India). All newly generated sequences in this study were deposited in GenBank (https://www.ncbi.nlm.nih.gov/genbank/) and listed in Table 3.

**Table 2.** Information on loci and primers used in this study.

Loci	PCR primers (forward/reverse)	Primer sequences	References
ITS	ITS1	TCCGTAGGTGAACCTGCGG	White et al. (1990)
	ITS4	TCCTCCGCTTATTGATATGC	
LSU	LR0R	ACCCGCTGAACTTAAGC	Vilgalys and Hester (1990)
	LR3	CCGTGTTTCAAGACGGG	

**Table 3.** Names, voucher numbers, country of origin, and corresponding GenBank accession numbers of the taxa used in the phylogenetic analysis.

Taxon name	Specimen voucher	Country of origin	GenBank accession no.		References
			nrITS	nrLSU	
***Volvariella areolavolvata***	**CAL 2383^T^**	**India**	PX776811	**PX764990**	**This study**
***V. areolavolvata***	**CAL 2384**	**India**	**PX776812**	**PX764991**	**This study**
*V. bilobata*	CUH AM778	India	ON922568	**-**	Chattopadhyay et al. (2022)
*V. bilobata*	GUBH 19922	India	ON922564	**-**	Chattopadhyay et al. (2022)
*V. bombycina*	AJ244	Spain	HM562212	HM562256	Justo et al. (2011a)
*V. bombycina*	SDBR-CMUNK0726	Thailand	OM417508	OM373644	Kumla et al. (2022)
*V. caesiotincta*	MA54717	Spain	HM562211	HM562255	Justo et al. (2011a)
*V. cf. sathei*	Hama390	Niger	KF926666	**-**	Daniëls et al. (2015)
*V. cubensis*	ARF-4948	USA	OP580290	**-**	GenBank
*V. diplasia*	CBS 355.64	India	MH858454	MH870086	Vu et al. (2019)
*V. dunensis*	JAC10587	New Zealand	MN738630	**-**	Unpublished
*V. dunensis*	SCM3513	Spain	JF415140	**-**	Vizzini et al. (2011)
*V. hypopithys*	TO AV137	Italy	HM246492	HM246488	Unpublished
*V. krizii*	PR1516257	Czech Republic	**-**	MK770133	Kaygusuz et al. (2020)
*V. lanata*	LE F-347402	Russia	OR872182	**-**	Malysheva and Malysheva (2024)
*V. lanata*	LE F-347403	Russia	OR872183	**-**	Malysheva and Malysheva (2024)
*V. lepiotospora*	AJ155	USA	HM562214	HM562259	Justo et al. (2011a)
*Vp. earlei*	TO HG2001	Italy	HM246498	HM246477	Justo et al. (2011b)
*V. morozovae*	LE 313229	Vietnam	MF377507	MF377508	Crous et al. (2017)
*V. morozovae*	LE 313322	Vietnam	MK882994	**-**	Crous et al. (2017)
*V. murinella*	GLM-F43392	Germany	MK412361	**-**	Unpublished
*V. murinella*	GLM-F42624	Germany	MK412400	**-**	Unpublished
*V. murinella*	G0742	Hungary	**-**	MK278657	Varga et al. (2019)
*V. neovolvacea*	SDBR-CMUNK0758	Thailand	OM417503	OM373653	Kumla et al. (2022)
*V. neovolvacea*	SDBR-CMUNK0760	Thailand	OM417505	**-**	Kumla et al. (2022)
*V. nivea*	GDGM 25489	China	FJ749127	**-**	Li et al. (2009)
*V. nullicystidiata*	SP393639	Brazil	EU920671	EU920670	Menolli Jr and Capelari (2008)
*V. orientalis*	LE 313241	Vietnam	MK882999	**-**	Malysheva et al. (2019)
*V. orientalis*	LE 313654	Vietnam	OL415619	**-**	Malysheva et al. (2019)
*V. pulla*	LE 313325	Vietnam	MK882998	MK883003	Malysheva et al. (2019)
*V. pusilla*	TO AV139	Italy	HM246494	HM246479	Unpublished
*V. reidii*	FI-1.2	Finland	MK770140	**-**	Kaygusuz et al. (2020)
*V. reidii*	FI-1.1	Finland	MK770139	**-**	Kaygusuz et al. (2020)
*V. rostricystidiata*	MFLU19-1528	Thailand	MT074694	MT078695	Niego et al. (2021)
*V. rostricystidiata*	MFLU19-1531	Thailand	MT074695	MT078696	Niego et al. (2021)
*V. sathei*	AMH 9436	India	JN792550	**-**	Senthilarasu et al. (2012)
*V. strangulata*	TO AV141	Italy	HM246493	HM246484	Unpublished
*V. surrecta*	AJ55	Spain	HM562213	HM562254	Justo et al. (2011a)
*V. surrecta*	GLM-F61563	Germany	MK412358	**-**	Unpublished
*V. surrecta*	G0848	Hungary	**-**	MK278659	Unpublished
*V. taylorii*	AJ54	Portugal	HM562210	HM562260	Justo et al. (2011a)
*V. terrea*	LUG11010	France	JF415141	**-**	Vizzini et al. (2011)
*V. terrea*	K(M):195631	United Kingdom	MZ159529	**-**	Unpublished
*V. thailandensis*	SDBR-CMUNK0957	Thailand	OM417504	**-**	Kumla et al. (2022)
*V. volvacea*	JAC12235	New Zealand	MN738642	MN738572	Unpublished
*V. volvacea*	TO AV143	Slovenia	HM246500	HM246486	Unpublished
*Volvopluteus asiaticus*	TNSF15191	Japan	HM562206	PQ268960	Justo et al. (2011b)
*Vp. asiaticus*	LE F-332246	Russia	OP862868	OP862870	Kalinina and Malysheva (2023)
*Vp. asiaticus*	HD2022	China	OP599925	**-**	Unpublished
*Vp. asiaticus*	HD2021	China	OP597804	**-**	Unpublished
*Vp. canalipes*	UES 10301	Mexico	MW743282	MW743277	Montoya et al. (2021)
*Vp. earlei*	TO AV133	Italy	HM246496	HM246480	Justo et al. (2011b)
*Vp. deliciosus*	GDGM73195	China	MK944281	MN056508	Li et al. (2025a)
*Vp. deliciosus*	GDGM74751	China	MK944280	MN056509	Li et al. (2025a)
*Vp. earlei*	LAH35715	Pakistan	MW362280	**-**	Unpublished
*Vp. earlei*	OKA TR654	Turkey	MW033394	MW029825	Unpublished
*Vp. earlei*	Mamet7	Democratic Republic of Congo	HM562205	MK278661	Justo et al. (2011b)
*Vp. earlei*	MA22816	Spain	HM562204	**-**	Justo et al. (2011a)
*Vp. earlei*	F14/10	Australia	OP809557	**-**	Unpublished
*Vp. earlei*	F86/11	Australia	OP809558	**-**	Unpublished
***Vp. fibrillobrunneus***	**CAL 2385 T**	**India**	**PX764988**	**PX764992**	**This study**
***Vp. fibrillobrunneus***	**CAL 2386**	**India**	**PX764989**	**PX764993**	**This study**
*Vp. gloiocephalus*	TO AV136	Italy	HM246495	HM246478	Vizzini et al. (2011)
*Vp. gloiocephalus*	TO AV135	Italy	HM246490	HM246476	Vizzini et al. (2011)
*Vp. gloiocephalus*	AFTOL-ID 890	USA	DQ494701	**-**	Matheny et al. (2006)
*Vp. gloiocephalus*	OKA-TR659	Turkey	MW033399	MW029830	Unpublished
*Vp. gloiocephalus*	OKA-TR660	Turkey	MW033400	MW029831	Unpublished
*Vp. gloiocephalus*	OKA TR665	Turkey	MW033405	**-**	Unpublished
*Vp. gloiocephalus*	AJ239	Spain	HM562202	MK278662	Justo et al. (2011a)
*Vp. gloiocephalus*	CM042	Algeria	KP826741	**-**	Unpublished
*Vp. gloiocephalus*	LOU18247	Spain	HM562209	**-**	Justo et al. (2011b)
*Vp. gloiocephalus*	NLB 1579	Australia	OP809559	**-**	Unpublished
*Vp. gloiocephalus*	PBM2272	USA	HM562203	**-**	Justo et al. (2011a)
*Vp. longipes*	HKAS135864	China	PQ451543	**-**	Zhu et al. (2025)
*Vp. longipes*	HKAS135865	China	PQ451544	**-**	Zhu et al. (2025)
*Vp. longipes*	HKAS135863	China	PQ451542	**-**	Zhu et al. (2025)
*Vp. michiganensis*	Smith32-590	USA	HM562195	**-**	Justo et al. (2011a)
*Vp. michiganensis*	LE 312006	Russia	MK729542	**-**	Zheng et al. (2025)
*Vp. michiganensis*	LE 311991	Russia	MK049912	**-**	Zheng et al. (2025)
*Vp. michiganensis*	HMJAU-CR106	China	MW242669	**-**	Zheng et al. (2025)
*Vp. michiganensis*	UBC F-32158	Canada	MF954699	**-**	Zheng et al. (2025)
*Vp. michiganensis*	LE311989	Russia	MK729541	**-**	Zheng et al. (2025)
*Vp. platensis*	HKAS135867	China	PQ451546	**-**	Zhu et al. (2025)
*Vp. platensis*	HKAS135866	China	PQ451545	**-**	Zhu et al. (2025)
*Vp. yunnanensis*	GMB-W1183	China	PQ764862	**-**	Zheng et al. (2025)
*Vp. yunnanensis*	GMB-W1187	China	PQ809775	**-**	Zheng et al. (2025)
*Cantharocybe gruberi*	AH24539	Spain	JN006422	**-**	Malysheva et al. (2019)

**Notes**: The newly generated sequences in this study are in bold black; “T” indicates the holotype specimens, and “-” indicates unavailable sequences.

### Dataset representation

The amplified sequences were checked for quality, assembled using the BioEdit Sequence Alignment Editor version 7.2.5 (Hall 1999), and used to perform BLASTn searches against the NCBI GenBank database (https://blast.ncbi.nlm.nih.gov/Blast.cgi). Subsequently, closely related sequences of the taxa with zero E-values were retrieved from the database to generate the dataset. Additionally, the sequences used in earlier studies on *Volvariella* and *Volvopluteus* (Justo et al. 2011a; Malysheva et al. 2019; Chattopadhyay et al. 2022; Zheng et al. 2025) were also obtained from the database to prepare the final dataset (Table 3). The sequence of *Cantharocybe gruberi* has been used as an outgroup taxon as per the study by Malysheva et al. (2019).

### Sequence alignment and phylogenetic analyses

The combined nrITS and nrLSU datasets were aligned in the CIPRES XSEDE resource using the online program MAFFT v7.427 (Katoh and Standley 2016; Katoh et al. 2019). The aligned sequences were then imported into Aliview v1.17.1 (Larsson 2014) for manual improvement and trimming of both ends.

In the CIPRES web portal (http://www.phylo.org/portal2/) (Miller et al. 2012), jModelTest 2.1.10 v20160303 (Darriba et al. 2012) was used to identify a statistically suitable model for the given dataset to perform Maximum likelihood (ML) analysis. The GTR+I+G model was chosen for the dataset based on the lowest BIC value of 14452.082637.

**Figure 1.**
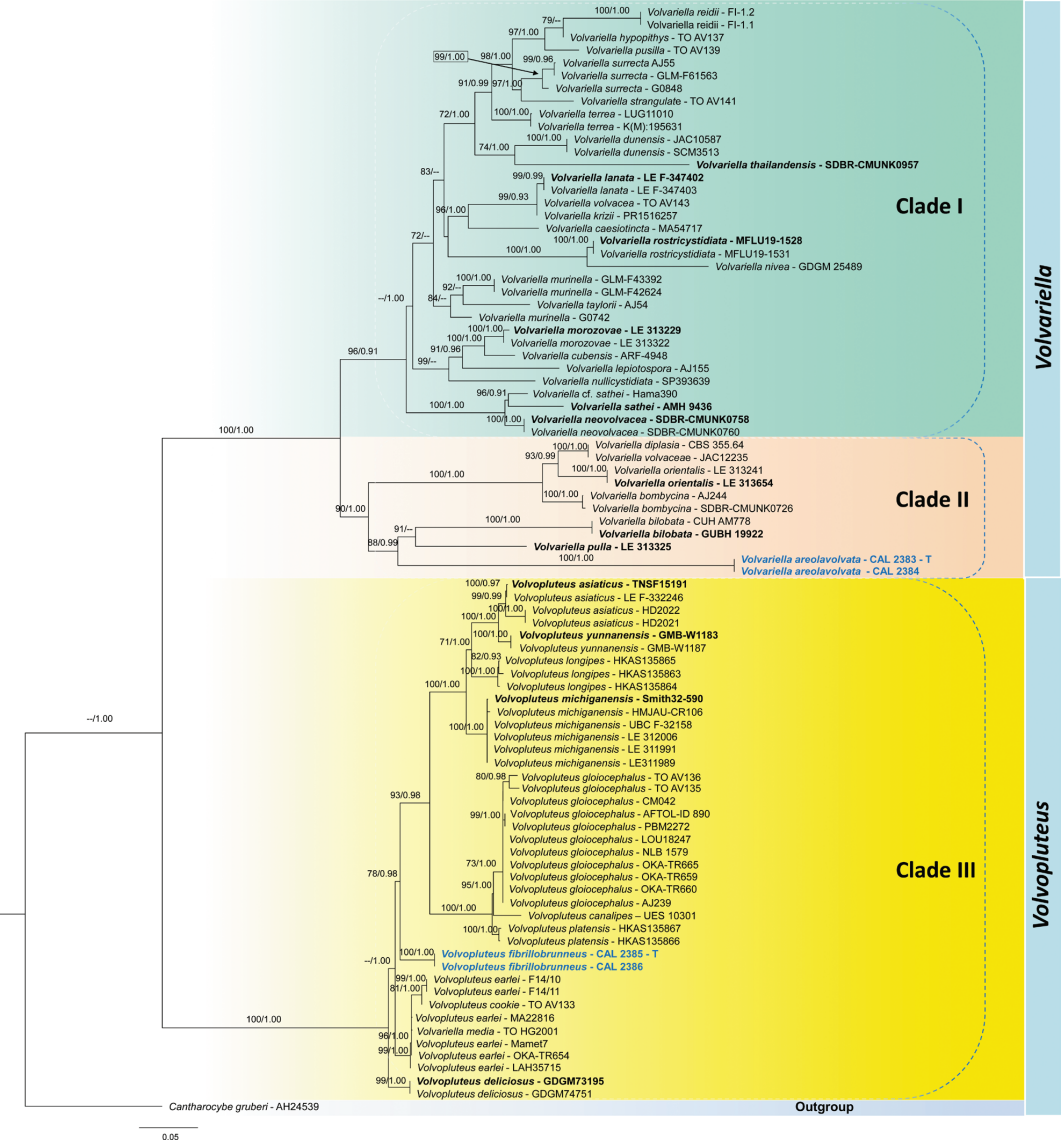
Phylogenetic tree for *Volvariella* and *Volvopluteus* generated from the combined nrITS and nrLSU rDNA sequences using maximum likelihood (−*InL* = 14452.082637) analysis and Bayesian inference (BI) analyses. Values to the left of ‘/’ are MLBS support, and those to the right of ‘/’ indicate Bayesian posterior probability (PP) support. The MLBS values ≥ 70% and PP values ≥ 0.90 are shown above or below the nodes. The newly generated sequences are shown in bold blue. Voucher numbers for the sequences are indicated in the tree after the taxon name, and other type species are indicated in bold black. ‘T’ denotes the holotype of the newly sequenced taxa.

Maximum likelihood analysis was performed with RAxML-HPC2 v8.2.12 (Stamatakis 2014) on the CIPRES XSEDE resource using the selected model from jModelTest 2.1.10 v20160303, with 1,000 bootstrap replicates. Bayesian analyses (BA) were conducted with MrBayes v3.2.2 (Ronquist et al. 2012) using MCMC methods (Geyer 1991) under a GTR+I+G model. Markov chains were run for 1 × 10^6^ generations, saving a tree every 1000^th^ generation, with all remaining parameters set to default. The average standard deviation of split frequencies reached 0.013 at the end of the run. Convergence was further assessed by examining effective sample size (ESS) values and potential scale reduction factor (PSRF) values. ESS values for all parameters exceeded 200, including the log-likelihood (lnL) with an ESS of 215, indicating adequate sampling of the posterior distribution. PSRF values for all parameters were close to 1.00, confirming convergence among independent runs. The initial 25% of recovered trees were excluded as burn-in, and the remaining trees were then used to estimate the posterior probabilities of the group. ML bootstrap values (MLBS) ≥ 50% and Bayesian posterior probability (PP) values ≥ 0.90 are reported in the resulting tree (Fig. 1). The phylogenetic trees were visualized and edited using FigTree v1.4.0 software (Rambaut and Drummond 2012).

## Results

### Phylogenetic analyses

The phylogenetic analyses were conducted using combined nrITS and nrLSU rDNA datasets. A total of 87 sequences were aligned, and the ends of each were subsequently trimmed. This resulted in a dataset consisting of 1,547 nucleotides; after trimming the ends of the individual alignments, the aligned datasets were 702 bp for nrITS and 845 bp for nrLSU, including gaps. The combined alignment comprised 649 parsimony-informative characters, 142 singleton sites, and 961 constant characters. The estimated base frequencies were as follows: A = 0.251, C = 0.215, G = 0.316, T = 0.218; substitution rates AC = 2.22735, AG = 5.33961, AT = 2.22735, CG = 1.00000, CT = 16.55070, GT = 1.00000. The phylogenetic tree derived from ML and MrBayes analyses exhibited nearly identical topology. Therefore, the tree obtained from ML analysis (−*InL* = 14452.082637) has been displayed in the present manuscript (Fig. 1), and the support values recovered from ML (MLBS ≥ 50%) and BA (PP ≥ 0.90) are presented.

In the phylogenetic tree (Fig. 1), taxa belonging to the genera *Volvariella* and *Volvopluteus* are separated from each other, and each genus appears monophyletic for the included taxa.

#### *Volvariella* s.str.

Members of the genus *Volvariella* are subdivided into two distinct clades (Clade I and Clade II), agreeing with Chattopadhyay et al. (2022). Clade I is well supported by the maximum likelihood bootstrap values (96% MLBS) but shows only moderate support in Bayesian posterior probability (0.91 PP) and includes all other *Volvariella* species. Clade II comprises taxa that include *V. diplasia*, *V. volvacea*, *V. orientalis*, and *V. bombycina*; in contrast, the remaining species of *V. bilobata*, originally reported from India, and *V. pulla*, recorded from Vietnam, together with the newly proposed species *Volvariella areolavolvata* (CAL 2383), are in this same clade, confirming its systematic position within the genus *Volvariella* (Fig. 1).

#### *Volvopluteus* s.str.

Phylogenetic analyses of the genus *Volvopluteus* (Clade III) are also statistically well supported (100% MLBS and 1.00 PP), and this clade includes all representative sequences of *Volvopluteus*, along with those of the newly described Indian taxon *Vp. fibrillobrunneus* (CAL 2385), confirming its systematic position within the genus *Volvopluteus* (Fig. 1). Moreover, *Vp. fibrillobrunneus* is further placed between *Vp. platensis* and *Vp. earlei* in the phylogenetic analysis, with moderate MLBS and strong PP support (78% MLBS/0.98 PP).

### Taxonomy

#### Volvoriella areolavolvata

Taxon classificationFungi

E. Tarafder, Enjam & A.R. Sherpa. sp. nov.

4024FB20-B0BB-549C-8CEF-ED5CB0B650BE

Index Fungorum: IF904792

Figs 2, 3

##### Etymology.

The specific epithet “*areolavolvata*” is derived from the Latin aeriolatus (areolate cracking on the outer surface) and refers to the characteristic of the volva.

##### Diagnosis.

*Volvariella areolavolvata* differs from *Volvariella bombycina* by a rigid volva with a distinctive externally areolate, cracked surface that forms pale to dark brown patches, slightly smaller basidiospores (6.8–9.0 × 4.4–5.5 μm), and larger cheilocystidia (63–131 × 21–37 μm).

##### Holotype.

India • West Bengal: North-24-Parganas District, Barasat, Talikhola, 22°44'50.9"N, 88°26'54.7"E, elev. 13.0 m, solitary on dead wood of *Ficus* sp., 07 August 2025, A.R. Sherpa, E. Tarafder & E. Hoque, Sherpa 01/2025 (CAL 2383, holotype).

##### Description.

***Basidiomata*** medium to large. ***Pileus*** 100–150 mm, fleshy, ovoid to subglobose when young, hemispherical to broadly campanulate, expanding obtusely umbonate; surface dry, sometimes viscid, covered with conspicuous silky fibrils or silky hairs; color white to whitish (10A1), often becoming slightly yellowish (4A5) in the center with age; margin entire. ***Context*** 10 mm thick at the disc, cream white (2A2). ***Lamellae*** free, 15–20 mm broad, crowded with lamellulae of different lengths; white when young, becoming pinkish brown (8A2–4) at maturity; edge concolorous, entire. ***Stipe*** 80–120 × 8–10 mm, central, cylindrical, curved at the base, solid; surface slightly pruinose, white (1A1) creamy, yellowish white (3B3), weakly hairy. ***Volva*** 25–30 mm broad, erect, rigid, thick, and fleshy, irregularly 5–6 lobbed; internally white (1A1), externally cracked areolate pale to dark brown (6F6–8) patches. Odor and taste indistinct.

**Figure 2.**
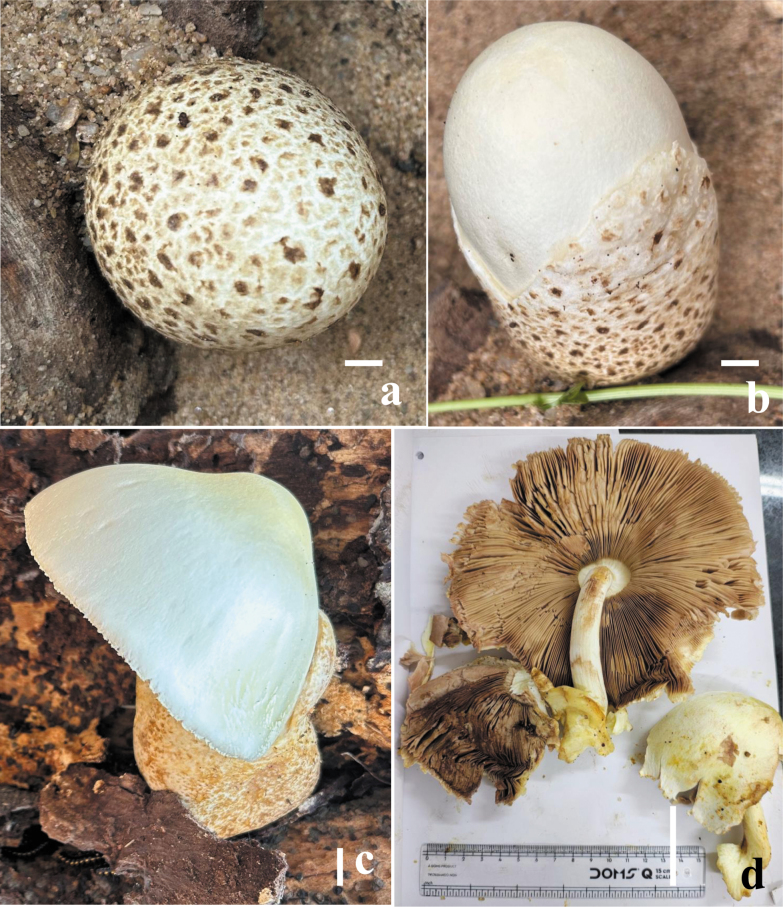
Field photographs of the basidiomata of (**a–d**) *Volvariella areolavolvata* (CAL 2383, holotype). Scale bars: 10 mm (**a–c**); 15 mm (**d**).

***Basidiospores*** [30/2/1] (6.8–)7.1–8.7(–9.0) × (4.4–)4.6–5.3(–5.5) µm, [X_mr_ = 7.61–7.92 × 4.85–4.95, X_mm_ = 7.75 ± 0.17 × 4.91 ± 0.33 μm, Q_mr_ = 1.23–1.85, Q_mm_ = 1.56 ± 0.18, n = 30 basidiospores per 2 specimens], oval to ellipsoid, hyaline to light yellowish in 5% KOH, smooth, dextrinoid, thin-walled, and apiculate. ***Basidia*** 17.4–26 × 7.4–10 µm, clavate, hyaline in 5% KOH, smooth, with 4 sterigmata, no clamp present at the base. ***Cheilocystidia*** 63–131 × 21–37 µm, hyaline in 5% KOH, thin-walled, mostly lageniform, with a long, narrow neck, forming a sterile layer at the lamellae edge. ***Pleurocystidia*** 44–56 × 13–17 µm, hyaline, thin-walled, broadly clavate with rounded apex or utriform, abundant in lamellae edge. ***Pileipellis*** a cutis made up of 10–37 µm broad, hyaline, thin-walled hyphae, clamp connections absent. ***Stipitipellis*** as compactly arranged cutis, 31–52 × 6.7–11.9 µm in diam., thin-walled, septate, clamp connections absent. ***Volva*** composed of septate, thin-walled 5.2–8.3 µm broad hyphae, with clamp connections absent.

**Figure 3.**
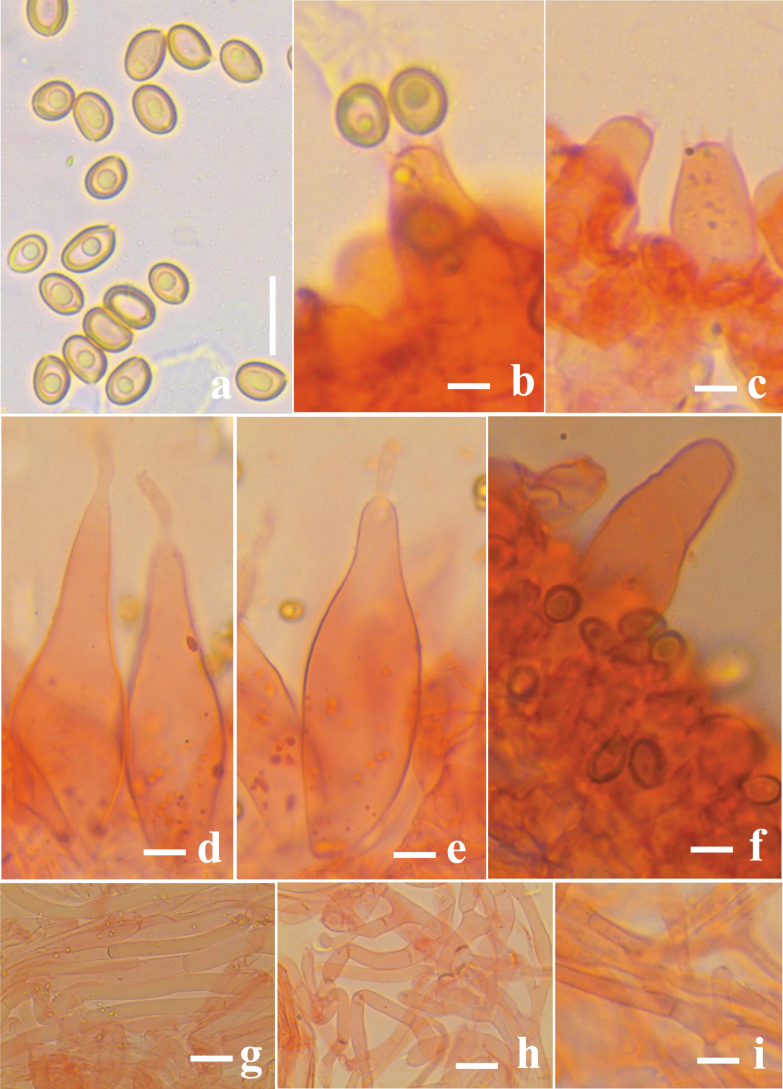
*Volvariella areolavolvata* (CAL 2383, holotype). **a**. Basidiospores; **b, c**. Basidia; **d, e**. Cheilocystidia; **f**. Pleurocystidia; **g**. Pileipellis hyphae; **h**. Stipe hyphae; **i**. Hyphal cells in the volva. Scale bars: 10 μm (**a**); 20 μm (**b–f**); 15 μm (**g–i**).

##### Habitat and known distribution.

Solitary, on dead wood of *Ficus* sp. So far, it is known only from India.

##### Additional specimens examined.

India • West Bengal: North-24-Parganas district, Barasat, Talikhola, 22°44'50.9"N, 88°26'54.7"E, elev. 13.0 m, 12 August 2025, A.R. Sherpa, E. Tarafder & E. Hoque, Sherpa 02/2025 (CAL 2384, paratype).

##### GenBank numbers.

CAL 2383 (holotype): nrITS = PX776811, nrLSU = PX764990. CAL 2384: nrITS = PX776812, nrLSU = PX764991.

##### Notes.

*Volvariella areolavolvata* is morphologically close to *Volvariella bombycina* but can be distinguished by a combination of macroscopic and microscopic characters. *Volvariella areolavolvata* is characterized by comparatively large basidiomata, a white to creamy pileus lacking squamules, a pure white stipe, and a white volva with brown patches, in combination with medium-sized basidiospores (mean = 7.75 × 4.9 µm). In contrast, *V. bombycina* differs by its robust and stout basidiocarps, a light-colored to pale brownish pileus that is entirely squamulose, a thick stipe, and a large, brown volva (Fig. 2). Moreover, *V. bombycina* has distinctive microscopic characteristics, including relatively small cheilocystidia (50–110 × 10–25 μm; Table 4) with frequently elongated apices.

**Table 4.** Comparison of *Volvariella areolavolvata* (CAL 2383) with its closely related taxa.

Name of the taxon	Pileus color	Pileus diameter (mm)	Stipe (mm)	Basidiospore (µm)	Basidia (µm)	Pleurocystidia (µm)	Cheilocystidia (µm)	Reference
***Volvariella areolavolvata***	**White to whitish, often becoming slightly yellowish in the center**	**100–150**	**80–120 × 8–10**	**6.8–9.0 × 4.4–5.5; Q = 1.23–1.85**	**17.4–26 × 7.4–10**	**44–56 × 13–17**	**63–131 × 21–37**	**This study**
*V. bombycina*	Whitish to pale ochraceous	70–190	70–190 × 7–20	6.5–9.5 × 4.0–6.5; Q = 1.20–1.70	20–30 × 8–11	40–100 × 10–20	50–110 × 10–25	Singer and Digilio (1951)
*V. biolobata*	Entirely grayish brown pileus	45–70	45–60 × 4–10	4.8–5.5 × 2.7–3.5; Q = 1.57–1.60	20–23 × 5–7	18.5–27.5 × 7–10	32–83 × 13–30	Chattopadhyay et al. (2022)
*V. pulla*	Pileus grayish brown or ash brown	40–75	55–70 × 3–5	5.5–7.0 × 4.0–5.0; Q = 1.20–1.59	15–22 × 7–8	52–83 × 14–34	45–75 × 14–23	Malysheva et al. (2019)

In our phylogenetic analyses, our collections (CAL 2383 and CAL 2384) clustered closely with *Volvariella pulla* and *Volvariella bilobata* but formed a distinct, well-supported clade (100% MLBS/1.00 PP; Fig. 1). *Volvariella pulla*, originally described from Vietnam (Malysheva et al. 2019), has an ochraceous or grayish brown stipe, an irregularly lobed volva colored dirty grey-brown with rusty brown spots, smaller basidiospores (5.5–8.0 × 4–5 μm vs. 6.8–9.0 × 4.4–5.5 μm), and much larger pleurocystidia (52–83 × 14–34 μm). The Indian collection of *Volvariella bilobata* differs from *V. areolavolvata* by its smaller pileus (45–70 mm diam.), colored grayish to dark brown, bilobed saccate volva, and much smaller basidiospores (4.8–5.5 × 2.7–3.5 μm; Fig. 3) and pleurocystidia (18.5–27.5 × 7–10 μm) (Chattopadhyay et al. 2022). Here, we introduce our collections as a novel species of *V. areolavolvata* based on morpho-molecular analyses.

#### Volvopluteus fibrillobrunneus

Taxon classificationFungiAgaricalesPluteaceae

Enjam, E. Tarafder & A.R. Sherpa. sp. nov.

4795E1CE-F5EA-54C1-B191-12D362BE9128

Index Fungorum: IF904793

Figs 4, 5

##### Etymology.

The specific epithet “*fibrillobrunneus*” refers to the brown color with a fibrillose pileus surface.

##### Diagnosis.

*Volvopluteus fibrillobrunneus* differs from *Volvopluteus earlei* by its fibrillose brown pileus surface with larger basidiocarps (Pileus < 50 mm in diameter *Vp. earlei*) and smaller basidiospores (8.3–11.5 × 5.1–7.1 μm).

##### Holotype.

India • West Bengal: North-24-Parganas district, Barasat, Near Kazibari Bus Stand, 22°44'45.5"N, 88°26'41.9"E, elev. 13.0 m, Scattered on the soil around with rich humus in broad-leaved forest, 18 September 2024, E. Hoque, AE-37/2024 (CAL 2385, holotype).

##### Description.

***Basidiomata*** medium to large. ***Pileus*** 80–97 mm diam., plano-convex to plane with age, low umbo at center, not viscid, dry, glabrous, innately radially fibrillose, striate, surface light brown (6D4), grayish brown (6D3) with grey (5B2) when young, dark blonde (5D4) center and birch grey (5C2) sides after maturity, becoming lighter grayish brown (5F3–5E4) towards the margin and yellowish brown (5B2–5C4) after dried; margin striate, radially fibrillose, slightly cracked with maturity, cream whitish squamulose present. ***Context*** 4 mm broad, white to grayish white (2B1). ***Lamellae*** free, approximately 10 mm broad, close to crowded, pinkish white (8A3–8B3), brownish gray (11C2) at maturity. ***Stipe*** 120–140 × 10–14 mm, central, cylindrical, solid, white, glabrous, not viscid, when young visibly broadening towards the base and in maturity with bulbous base enclosed in sac-like volva. ***Volva*** 15–19 × 8–10 mm, free from the stipe, saccate, membranous, 2–3 lobed, with a white to light brownish outer surface and whitish inner surface. Odor and taste indistinct.

**Figure 4.**
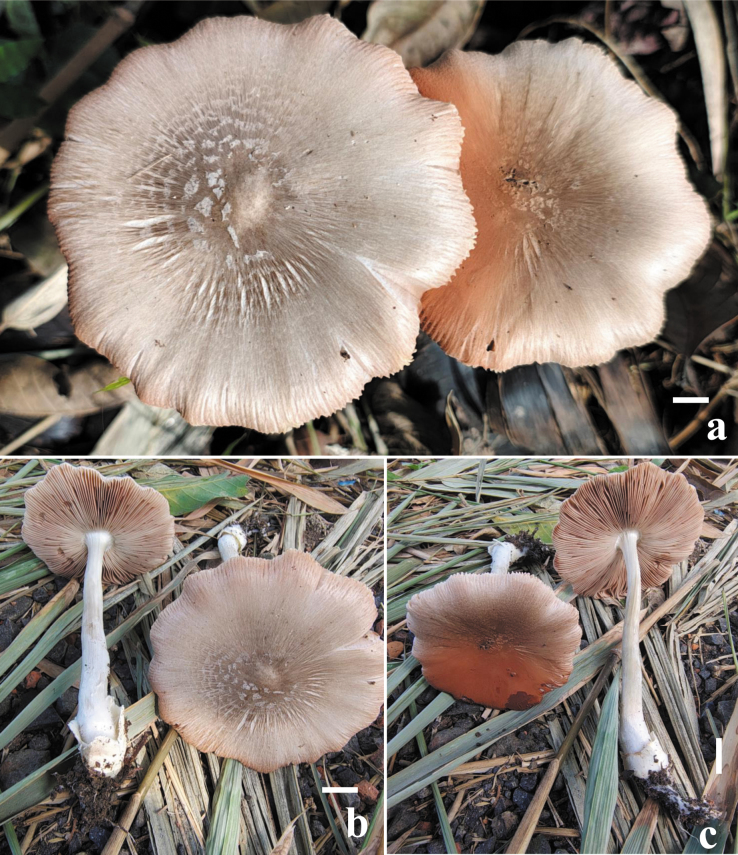
Field photographs of the basidiomata of (**a–c**) *Volvopluteus fibrillobrunneus* (CAL 2385, holotype) in natural habitat. Scale bars: 10 mm (**a–c**).

***Basidiospores*** (8.3–)8.5–9.6(–11.5) × (5.1–)5.4–6.3(–7.1) µm, [X_mr_ = 8.65–9.43 × 5.88–6.25, X_mm_ = 9.85 ± 0.92 × 6.30 ± 0.49 μm, Q_mr_ = 1.36–1.75, Q_mm_ = 1.57 ± 0.19, n = 30 basidiospores per 2 specimens], ellipsoid to elongate, smooth, hyaline to light yellowish in 5% KOH, thin-walled, and apiculate. ***Basidia*** 25.7–45.6 × 9.8–11.9 µm, clavate, hyaline in 5% KOH, smooth, with 2–4 prominent sterigmata, no clamp at base. ***Cheilocystidia*** 34–72 × 15–29 µm, hyaline in 5% KOH, thick-walled, mostly clavate, forming a sterile layer at the lamellae edge. ***Pleurocystidia*** 53–75 × 14–21 µm, hyaline, thin-walled, lageniform rostrate with apical appendages. ***Pileipellis*** ixocutis, made up of hyphae 25–32 µm broad, hyaline, thin-walled, clamp connections absent. ***Stipitipellis*** as compactly arranged hyphae, 23–38 µm broad, thin-walled, clamp connections absent. ***Volva*** composed of septate interwoven cylindrical hyphae, thin-walled 9–13 µm broad, clamp connection absent.

**Figure 5.**
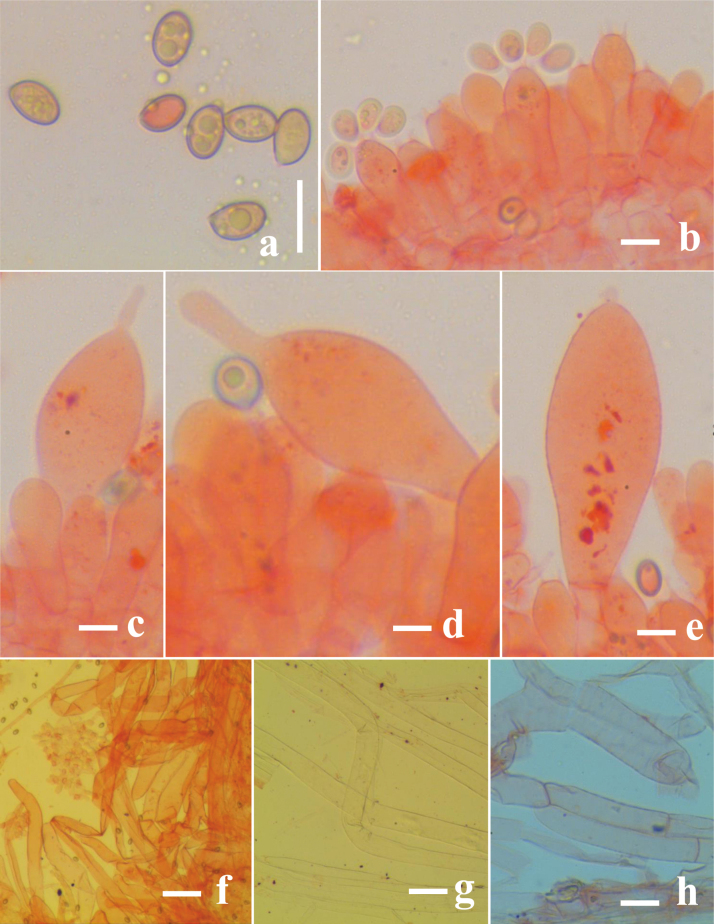
*Volvopluteus fibrillobrunneus* (CAL 2385, holotype). **a**. Basidiospores; **b**. Basidia; **c, d**. Cheilocystidia; **e**. Pleurocystidia; **f**. Pileipellis hyphae; **g**. Stipe hyphae; **h**. Hyphal cells in the volva. Scale bars: 10 μm (**a**); 20 μm (**b–e**); 15 μm (**f–i**).

##### Habitat and known distribution.

Scattered on the soil around with rich humus in a broad-leaved forest. So far, it is only known from India.

##### Additional specimens examined.

India • West Bengal: North-24-Parganas district, Barasat, near Kazibari Bus Stand, 22°44'45.5"N, 88°26'41.9"E, elev. 13.0 m, scattered on the soil around with rich humus in broad-leaved forest, 21 September 2024, E. Hoque, AE-41/2024 (CAL 2386, paratype).

##### GenBank numbers.

CAL 2385 (holotype): nrITS = PX764988, nrLSU = PX764992; CAL 2386: nrITS = PX764989, nrLSU = PX764993.

##### Notes.

*Volvopluteus fibrillobrunneus* is characterized by a medium- to moderately large-sized (80–97 mm) pileus, entirely light brown to grayish brown, with a radially fibrillose surface; close to crowded lamellae colored pinkish white to brownish gray; a multi-lobed saccate volva with a white to light brownish outer surface and a whitish inner surface (Fig. 4); larger basidiospores measuring 8.3–11.5 × 5.1–7.1 μm; clavate to ventricose cheilocystidia measuring 34–72 × 15–29 μm; lageniform rostrate pleurocystidia with apical appendages measuring 53–75 × 14–21 μm; and habitat on rich humus soil (Fig. 5). Among macro-morphologically similar taxa (Table 5), *Vp. earlei* can be distinguished by its smaller basidiospores (11–16 × 8–11 μm), the presence of longer pleurocystidia (up to 110 μm), and the presence of cheilocystidia (Shaffer 1962; Justo and Castro 2010; Giannoni et al. 2018). Moreover, *Volvopluteus earlei* was originally described from Cuba (Murrill 1911) and later reported from the USA (Coker 1947), Mexico (Vázquez et al. 1989), Africa (Heinemann 1975), Argentina (Niveiro and Albertó 2012), Italy (Contu 2006; Giannoni et al. 2018), Spain (Justo and Castro 2010), Turkey (Kaygusuz et al. 2021), and India (Chouhan and Panwar 2021).

**Table 5.** Comparison of *Volvopluteus fibrillobrunneus* (CAL 2385) and its closely related taxa. Abbreviations: NR = not reported.

Name of the taxon	Pileus color	Pileus diameter (mm)	Stipe (mm)	Basidiospore (µm)	Basidia (µm)	Pleurocystidia (µm)	Cheilocystidia (µm)	Reference
***Volvopluteus fibrillobrunneus***	**Light brown to gaeyish brown when young, dark blonde center and birch grey sides after maturity**	**80–97**	**120–140 × 10–14**	**8.3–11.5 × 5.1–7.1**	**25.7–45.6 × 9.8–11.9**	**53–75 × 14–21**	**34–72 × 15–29**	**This study**
*Vp. earlei*	White or ochraceous buff, light ochraceous at center	25–45	35–65 × 3–7	11.0–16.0 × 7–9.5	30–47 × 10–18	47–98 × 14–20	25–50 × 10–35	Montoya et al. (2021)
*Vp. deliciosus*	Surface usually grayish	50–110	80–140 × 8–27	12.5–16.5 × 7.5–10	42–65 × 13–16	62–88 × 12–33	50–65 × 18–32	Li et al. (2025a)
*Vp. diversisporus*	Surface orange white	41–59	36–85 × 4–6	11.2–16.0 × 7.2–14.4	25.6–46.4 × 9.6–17.6	20.8–86.4 × 8.8–34.0	32.0–80.0 × 9.6–40.0	Kaur and Singh (2014)
*Vp. earlei*	Slightly darker or pale yellowish biscuit in center	20–50	40–70 × 5–10	12–15.5 × 7.3–9.5	35–51 × 10–14	31–85 × 15–38	30–75 × 11–24	Bougher and Barrett (2023)
*Vp. gloiocephalus*	Uniformly grayish beige except darker brownish at the umbo	30–110	35–120 × 5–22	12–17.5 × 7.5–10	42–56 × 12–16	45–60 × 20–40	32–70 × 10–25	Bougher and Barrett (2023)
*Vp. longipes*	Slightly brownish at center, grayish white to dirty white	48–70	130–150 × 4–10	14–20 × 8–11	35–60 × 16–23	NR	40–75 × 16–29	Zhu et al. (2025)
*Vp. michiganensis*	Gray to brownish gray	70–90	80–110 × 10–15	10.5–13.5 × 6.5–8	35–55 × 10–15	70–110 × 25–45	60–75 × 15–27	Justo et al. (2011a)
*Vp. platensis*	Grayish white to dirty white, sometimes with a cream tinge	30–45	50–80 × 4–10	15.5–20 × 9–12	35–68 × 15–20	65–95 × 18–40	NR	Zhu et al. (2025)
*Vp. shafferii*	Surface grayish orange, orange grey at center, with pinkish tinge towards margin	10–112	70–165 × 18–25	11.2–16 × 7.2–12.8	17.6–34.0 × 8.9–16.0	NR	NR	Kaur and Singh (2014)
*Vp. striatellus*	Pale grey when young, transitioning to white with grayish tints at maturity	48–80	110–160 × 5–23	15.5–18.8 × 9.6–11.5	40.8–55 × 15.1–19.9	62–113.8 × 24.5–48.5	50.1–81.5 × 24.1–34.9	Li et al. (2025b)
*Vp. yunnanensis*	Dark blonde center and birch grey sides	59–85	120–137 × 11–13	11.3–12.9 × 6.0–6.9	43–54 × 10–15	60–82 × 16–25	60–86 × 14–27	Zheng et al. (2025)

In the phylogenetic analyses (Fig. 1), *Vp. fibrillobrunneus* is placed as a sister group to *Vp. platensis*, originally described from China (Zhu et al. 2025), and can be distinguished from *Vp. fibrillobrunneus* by the smaller basidiomata (48–70 mm broad in *Vp. platensis*), a slightly brownish to grayish white pileus, and clavate to fusoid cheilocystidia (Zhu et al. 2025). Hence, here we introduce our collections as a novel species of *Volvopluteus* s.str., i.e., *Vp. fibrillobrunneus*, based on morpho-molecular analyses.

## Discussion

Geographically, West Bengal is located in eastern India and is bordered by the high Himalayan peaks in the north, coastal ecosystems in the south, and the Gangetic delta and plateaus in between (Tarafder et al. 2023). Subtropical broadleaved to subalpine forests dominate in the Himalayan region; in contrast, littoral and swamp forests cover the coastal regions, and dry deciduous forests prevail on the plateaus and in the Gangetic plains (Dutta et al. 2015). This wide range of phyto-topographical features, together with altitudinal and climatic variations, creates ample opportunities for the luxuriant growth of macrofungi. Although several species of *Volvariella* have been reported from West Bengal, India, most earlier studies were based solely on morphological characterization, viz., *Volvariella castanea* (Massee) G.C. Rath (Calcutta, Massee 1912), *V. delicatula* (Massee) Manjula (Calcutta, Massee 1912), *V. diplasia* (Berk. & Broome) Singer (Hooghly and Bose 1920a), *V. pusilla* (Pers.) Singer (South 24-Parganas ‘fide’ Dutta et al. 2011, 2013), *V. terastia* (Berk. & Broome) Singer (Berk. & Broome) Singer (Calcutta, Bose 1920b, 1921), *V. thwaitesii* (Hook. f. ex Berk.) G.C. Rath (Darjeeling, Berkeley 1850), and *V. volvacea* (Bull.) Singer (South 24-Parganas ‘fide’ Dutta et al. 2011, 2013).

*Volvariella areolavolvata* was characterized by a large-sized (100–150 mm), entirely white pileus with a slightly silky hairy surface, often becoming slightly yellowish in the center with age; crowded lamellae colored pinkish white to grayish brown; a volva that is rigid, thick, and fleshy, irregularly lobed, internally white, and externally cracked, with areolate pale to dark brown patches; smaller basidiospores (6.8–9.0 × 4.4–5.5 μm); mostly lageniform cheilocystidia (63–131 × 21–37 μm); broadly clavate with a rounded apex or utriform pleurocystidia (44–56 × 13–17 μm; Table 4); and habitat on dead wood. In our phylogenetic analyses, *V. areolavolvata* clustered closely with *V. pulla* and *V. bilobata*, forming a distinct and well-supported clade (100% MLBS/1.00 PP; Fig. 1).

Additionally, *Vp. fibrillobrunneus* falls between *Vp. platensis* and *Vp. earlei* in the phylogenetic analysis, with moderate MLBS and strong PP support (78% MLBS/0.98 PP). However, *Volvopluteus fibrillobrunneus* was characterized by a medium- to moderately large-sized (80–97 mm), entirely light brown to grayish brown pileus with a radially fibrillose surface; close to crowded lamellae colored pinkish white to brownish gray; a multi-lobed saccate volva with a white to light brownish outer surface and a whitish inner surface; larger basidiospores (8.3–11.5 × 5.1–7.1 μm); clavate to ventricose cheilocystidia (34–72 × 15–29 μm); and lageniform pleurocystidia (55–60 × 29–47 μm). Among macro-morphologically similar taxa (Table 5), *Vp. earlei* can be distinguished by its smaller basidiospores (11–16 × 8–11 μm) and the presence of longer pleurocystidia (up to 110 μm). In the phylogenetic tree for the combined dataset (Fig. 1), our newly described taxon is also close to *Vp. platensis*, which was initially described by relatively small basidiomata, with pileus diameters not exceeding 45 mm, a slightly brownish to grayish white pileus, and clavate to fusoid cheilocystidia (Zhu et al. 2025).

The discovery of *V. areolavolvata* and *Vp. fibrillobrunneus* increases our understanding of the diversity and distribution of these genera in eastern India. In conclusion, *V. areolavolvata* and *Vp. fibrillobrunneus* are supported by both morphological and phylogenetic analyses. Moreover, their discovery significantly advances the understanding of agaric diversity in eastern India and underscores the need for continued exploration and integrative taxonomic studies, given modern phylogenetic analyses in the region.

## Supplementary Material

XML Treatment for Volvoriella areolavolvata

XML Treatment for Volvopluteus fibrillobrunneus

## Additional information

### Use of AI

No use of AI was reported.

### Author ORCIDs

Entaj Tarafder https://orcid.org/0000-0002-3680-3433

Enjamul Hoque https://orcid.org/0009-0004-3518-4839

Ang Rinzing Sherpa https://orcid.org/0000-0003-1488-3513

Samantha C. Karunarathna https://orcid.org/0000-0001-7080-0781

Meimei Wang https://orcid.org/0000-0003-3747-5432

Nalin N. Wijayawardene https://orcid.org/0000-0003-0522-5498

Qiang Li https://orcid.org/0000-0002-9735-8214

Fuqiang Yu https://orcid.org/0000-0001-7931-080X

Dong-Qin Dai https://orcid.org/0000-0001-8935-8807

Wannapawn Watsuntorn https://orcid.org/0009-0002-3196-4753

### Data availability

Sequence data generated for the present study have been deposited in GenBank. The accession numbers are as follows: CAL 2383 (nrITS: PX776811; nrLSU: PX764990); CAL 2384 (nrITS: PX776812; nrLSU: PX764991); CAL 2385 (nrITS: PX764988; nrLSU: PX764992); CAL 2386 (nrITS: PX764989; nrLSU: PX764993). The data can be accessed at https://www.ncbi.nlm.nih.gov/genbank/. All data that support the findings of this study are available in the main text.
